# Cognitive Impairment After Resolution of Hepatic Encephalopathy: A Systematic Review and Meta-Analysis

**DOI:** 10.3389/fnins.2021.579263

**Published:** 2021-03-10

**Authors:** Óscar López-Franco, Jean-Pascal Morin, Albertina Cortés-Sol, Tania Molina-Jiménez, Diana I. Del Moral, Mónica Flores-Muñoz, Gabriel Roldán-Roldán, Claudia Juárez-Portilla, Rossana C. Zepeda

**Affiliations:** ^1^Laboratorio de Medicina Traslacional, Instituto de Ciencias de la Salud, Universidad Veracruzana, Xalapa, Mexico; ^2^Laboratorio de Neurobiología de la Conducta, Departamento de Fisiología, Facultad de Medicina, Universidad Nacional Autónoma de Mexico, Ciudad de Mexico, Mexico; ^3^Facultad de Biología-Xalapa, Universidad Veracruzana, Xalapa, Mexico; ^4^Instituto Interdisciplinario de Investigaciones de la Universidad de Xalapa, Xalapa, Mexico; ^5^Programa de Doctorado en Ciencias Biomédicas, Universidad Veracruzana, Xalapa, Mexico; ^6^Laboratorio de Biomedicina Integral y Salud, Centro de Investigaciones Biomédicas, Universidad Veracruzana, Xalapa, Mexico

**Keywords:** learning, cognition, liver disease, cirrhosis, impairment, psychometric test, liver transplant

## Abstract

Hepatic encephalopathy (HE) is one of the most disabling metabolic diseases. It consists of a complication of liver disease through the action of neurotoxins, such as excessive production of ammonia from liver, resulting in impaired brain function. Its prevalence and incidence are not well known, although it has been established that up to 40% of cirrhotic patients may develop HE. Patients with HE episodes display a wide range of neurological disturbances, from subclinical alterations to coma. Recent evidence suggests that the resolution of hepatic encephalopathy does not fully restore cognitive functioning in cirrhotic patients. Therefore, the aim of this review was to evaluate the evidence supporting the presence of lingering cognitive deficits in patients with a history of HE compared to patients without HE history and how liver transplant affects such outcome in these patients. We performed two distinct meta-analysis of continuous outcomes. In both cases the results were pooled using random-effects models. Our results indicate that cirrhotic patients with a history of HE show clear cognitive deficits compared to control cirrhotic patients (Std. Mean Difference (in SDs) = −0.72 [CI 95%: −0.94, −0.50]) and that these differences are not fully restored after liver transplant (Std. Mean Difference (in SDs) = −0.48 [CI 95%: −0.77, −0.19]).

## Introduction

Hepatic encephalopathy (HE), also known as portosystemic encephalopathy, is a reversible syndrome that occurs as a complication of liver disease involving impaired brain function ranging from subclinical alterations to coma (Yanny et al., 2019). To date, the acute prevalence and incidence of HE worldwide is not known, perhaps due to several causes such as different etiological factors, severity of the disease, and challenges in diagnosing minimal or sub-clinical HE (Raphael and Shali Matuja, 2016; Elsaid et al., 2020). However, it has been established that up to 40% of cirrhotic patients develop HE (Kornerup et al., 2018).

HE has been classified using several criteria. According to the American Association for the Study of Liver Disease (AASLD) (EASL Clinical Practice Guidelines for the management of patients with decompensated cirrhosis, 2018) and the European Association for the Study of the Liver (EASL) (EASL Clinical Practice Guidelines for the management of patients with decompensated cirrhosis, 2018) guidelines, in order to “ensure adequate patient management and standardized performance of observational studies and clinical trials,” four main classification axes should be used: (1) the underlying cause, (2) the severity of the disease manifestation, (3) the time course of the disease, and (4) the existence of precipitating factors (Elsaid et al., 2020). In relation to disease cause, HE is classified into three subtypes: Type A, caused by acute liver failure; Type B, due predominantly to portosystemic bypass or shunting; and Type C, produced by cirrhosis. The latter is the most common risk factor for HE (Table 1) (Kornerup et al., 2018). According to the severity of the manifestations, the classification of HE relies on two scales: the West Haven Criteria (WHC) and the International Society for Hepatic Encephalopathy and Nitrogen Metabolism (ISHEN) grade (Table 2). The WHC criteria comprises six stages: unimpaired or grade 0, minimal and grades I through IV. Unimpaired patients do not experience any clinical or subclinical signs, nor do they have a history of HE. The Minimal Hepatic Encephalopathy (MHE) is the mildest form of the disease, without any clinical evidence of mental changes, but cognitive dysfunction, characterized by attention deficit, working memory problems and defects in executive functions, commonly detected using psychometric tests. Grade I patients present behavioral changes but minimal changes in level of consciousness, and clinical manifestations (Table 2). From grades II until IV, both the clinical manifestations and the cognitive impairment progressively worsen, leading to coma. On the other hand, in the ISHEN scale, covert HE corresponds to minimal and Grade I HE of the WHC scale; whereas, overt HE (OHE) refers to grade II to IV. A third way to classify HE is the time course of the disease. In this regard, three subtypes have been considered: episodic, in which patients experience HE episodes at least 6 months apart; recurrent, in which episodes appear every 6 months or less; and persistent, where the patients experience HE continuously, having different degrees over time, behavioral modifications and with relapses of OHE bouts. Finally, the presence of precipitating factors leads to the classification of: spontaneous and precipitated. The main HE precipitating factors are: infections, electrolyte disorder, gastrointestinal bleeding, diuretic overdose, excessive protein intake, diarrhea, hypoxia, peripheral vasodilatation, dehydration, among others (Al Sibae and McGuire, 2009).

**Table 1:** HE classification according to the underlying disease.

**Type**	**Cause**
A	Acute liver failure
B	Predominantly to portosystemic bypass or shunting
C	Cirrhosis

**Table 2:** HE classification according to the severity of the manifestations.

**WHC**	**ISHEN**	**Criteria**
0 or unimpaired		No evidence or history of HE
Minimal	Covert	No clinically abnormalities detected
Grade I		Trivial lack of awareness Euphoria or anxiety Shortened attention span Impairment of addition or subtraction Altered sleep rhythm
Grade II	Overt	Lethargy or apathy Disorientation for time Obvious personality change Inappropriate behavior Dyspraxia Asterixis
Grade III		Somnolence to semistupor Responsive to stimuli Confused Gross disorientation Bizarre behavior
Grade IV		Coma

*HE, hepatic encephalopathy; ISHEN, International Society for Hepatic Encephalopathy and Nitrogen Metabolism; WHC, West Haven criteria*.

HE pathogenesis is not well-understood, although it is suspected to involve the action of neurotoxins, such as excessive production of ammonia from the liver, by decreasing its metabolism. Moreover, changes in brain energy metabolism, systemic inflammatory response, and alterations of the blood brain barrier have also been observed (Ferenci, 2017). This may leter lead to neuropsychiatric abnormalities, including changes of personality, consciousness, cognition, motor function, attention deficits, among others (García-García et al., 2018). Hence, the type of liver disease must be properly identified and treated to restore brain function, yet even in the mildest form of HE, the quality of life is diminished. Moreover, little is known about the residual consequences in the psychosocial (cognitive) aspects in those patients who recover from HE. Although initially thought to be fully reversible with treatment (Prasad et al., 2007), recent clinical and functional evidence has suggested persistent cognitive alteration after resolution of OHE (Umapathy et al., 2014; García-García et al., 2018). Moreover, even though liver transplantation (LT) seems to solve the metabolic damage, the degree to which cognitive as well as behavioral impairments are reversed remains controversial.

Here, we performed a systematic review of the different studies that characterize distinct types of cognitive impairments in cirrhotic patients, with and without MHE. We aim to draw some conclusions from these works that have used a wide variety of neuropsychological test batteries as well as brain function assessing techniques. Finally, we performed a meta-analysis of those studies that explored the relation between a history of resolved OHE and cognitive impairment in cirrhotic patients and whether such functions are improved by LT.

## Methods

### Search Strategy and Eligibility Criteria

We performed the present systematic review according to the PRISMA-P guidelines for systematic reviews (Moher et al., 2015). Literature was searched from 2010 to 2020; in the following databases: PubMed, Wiley, EBSCO, Science Direct, and Springer; using the keywords and search terms: “hepatic encephalopathy learning impairment” and “hepatic encephalopathy cognitive impairment,” “cognitive impairment, liver transplantation, hepatic encephalopathy.” Articles with full-text available that describe the relation between hepatic encephalopathy and learning and/or cognitive deficits or impairments as the main topic were included in the review. Articles written in English were considered. Only human studies were included. The articles were discarded if they met any of the following criteria: do not describe learning and/or cognitive impairment associated with HE, articles that validate methods, case-control studies, and those that use animal models. Also, we excluded reviews, encyclopedia entries, book chapters, conference abstracts, book reviews, case reports, editorials, minireviews, news, patent reports, and practice guidelines. We included retrospective of cohort (R), prospective of cohort (P), longitudinal (L), and cross-sectional (CS) studies. When unclear or unreported data were detected in the studies, efforts were made to contact the authors.

### Data Extraction

Two authors selected the articles independently by reading the titles and abstracts of the studies retrieved from the electronic searches, removing the studies that did not meet the inclusion criteria. Afterwards, two more authors independently read the full-texts and according to the eligibility criteria, made the selection of articles to be included in the systematic review. In case of disagreements, a third review author was consulted to help with the final decision. The Endnote reference manager was used to eliminate duplicated articles. The extraction of the information was conducted independently by two authors, and entered into two different spreadsheets, containing the following data items: goal, country, sex, treatment (when included), number of participants, cognitive tests, techniques for analysis, main findings, and reference.

### Assessment of Risk of Bias

The risk of bias, design, and methodological quality were assessed using the Newcastle-Ottawa Scale (NOS) for cohort studies (Lo et al., 2014). This scale includes eight items that evaluate selection, comparison, and outcome. For each item, a star was awarded (except for comparison that can receive up to two stars). Studies receiving at least six stars (maximum of nine) were classified as good quality.

### Aim and Scope

The aim of the present review was to evaluate the extent to which a previous episode of OHE has a residual impact on cognition, as measured by several neuropsychological test batteries, mostly Psychometric Hepatic Encephalopathy Score (PHES), and to assess whether this could be reversed by LT. For this purpose, we included studies where a group of cirrhotic patients with a history of at least one OHE episode was compared to a control group of cirrhotic patients with no history of OHE. Some of these studies assessed cognition on these two groups of patients after LT and were included in a subsequent analysis. Of those, three studies (Sotil et al., 2009; Garcia-Martinez et al., 2011; Hopp et al., 2019) were included only in the post-LT due to lack of sufficient pre-LT data concerning neuropsychological tests scores for patients with and without a history of OHE specifically.

### Data Synthesis and Meta-Analysis

#### Data Extraction

The neuropsychological test batteries used for cognitive function assessment varied considerably among studies but all of them included the “gold-standard” PHES or at least some of the tests included in the PHES. The results from PHES (or parts of it) were extracted from all the studies, most of which reported the sum of SDs *vs*. controls for the five PHES tests. All but two studies (Bajaj et al., 2011; Cheng et al., 2018) included all PHES tests. In cases where no summarized PHES score was reported, the mean differences ± SD between the results for each individual test in a given group of subjects vs. those of the controls were calculated and the average mean differences and SDs were obtained and included in the analysis. Also, when no control was included in the study, reference scores for each test were obtained according to the demographic characteristics of the patients, using previously published equations (Duarte-Rojo et al., 2011). When the PHEs summarized data was reported as median (IQR), we calculated the mean ± SD with the help of a previously published method (Hozo et al., 2005). Many studies did not report gender so data for both men and women were lumped together in the analysis.

We performed two distinct meta-analysis of continuous outcomes and, in both cases, the results were pooled using the inverse variance method in a random-effects model, given the apparent clinical, methodological, and statistical variations between studies. Standardized mean differences (SMDs) were obtained from the extracted data as effect sizes from each study and the outcomes were combined in a pooled analysis. The first analysis compared the HE and NHE groups in patients that did not receive LT (non-LT) while the second compared both groups at >6 months after LT (LT). In cases where patients were tested repeatedly, the mean scores were obtained from the first testing session, except for Bajaj et al. (2011) in which OHE patients had their first episode between the two-testing session, hence comparisons were made between the scores obtained in second sessions of each group.

The pooled size effects are presented as Z value and heterogeneity is presented as Chi-square tests results as well as *I*^2^ values, with <30% indicating low; 30–60% moderate; 60–90% substantial; and >90% considerable heterogeneity (Higgins et al., 2019). The meta-analysis and forest plots were performed using Review Manager (version 5.4; the Cochrane Collaboration). Reported *p*-values are two-sided.

## Results

### Description of the Studies Included in the Analytical Review and Meta-Analyses

**Figure 1:**
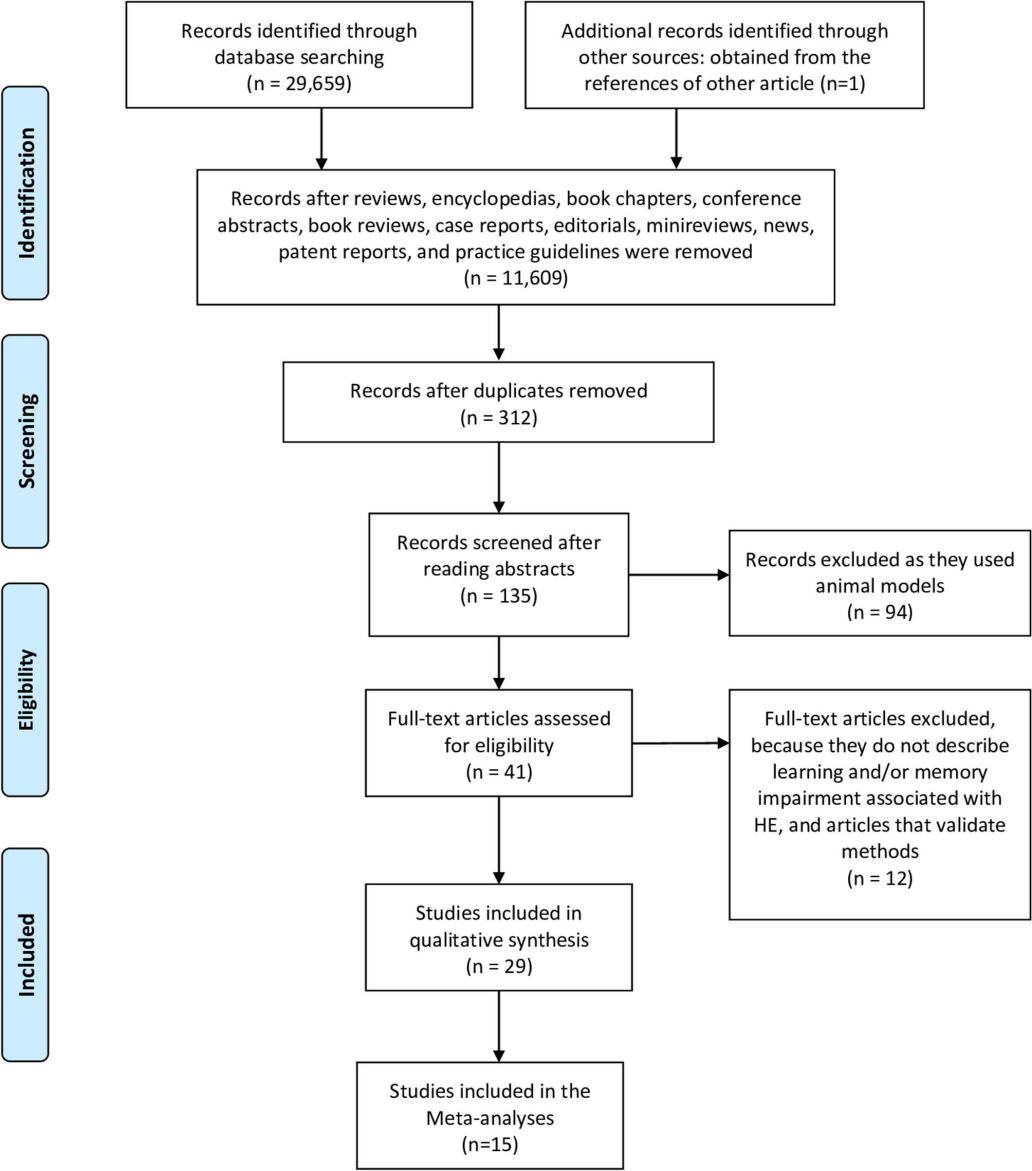
PRISMA 2009 flow diagram.

In Figure 1, we schematize the protocol of the searches and selection of articles according to the PRISMA flow chart. Using the methodology described in the Methods section, we initially identified 29,659 articles, in all the databases accessed, distributed as following: 274 from PubMed, 7,758 from Wiley, 72 from Web of Science, 8,348 from Science Direct, 7,206 from EBSCO, and 6,001 from Springer. After the elimination of reviews, book chapters, poster and conferences presentations, we obtained 11,609 records. Then, duplicates were removed, 135 abstracts were screened, 41 full-texts were read and 29 articles that fit the eligibility criteria were included in this review. One study (Sotil et al., 2009) included in the review was identified from the references of another article obtained in the original searches. Eleven studies used a prospective cohort study design, three used cross-sectional design, three were retrospective of cohort studies, seven were longitudinal study designs, and five used a mix design. Studies were conducted in 10 countries; two in China, two in Germany, two in Denmark, two were from Spain, one from India, seven from Italy, one from South Korea, two from Poland, and seven from United States. We built the searches from 2010 to 2020, however most of the studies included in this review were published in 2014 (6), following by 2011, 2017, and 2019 (4); in 2013, 2015, and 2018, two studies were published each year; and finally, in 2009, 2010, 2012, 2016, and 2020, one study was published each year.

In Supplementary Table 2, we summarize the main information extracted from the 29 studies included in this review and meta-analysis. All the studies were included since they fit all the criteria used in the searches, however, only those articles that fitted the meta-analysis criteria were used (see above).

### Meta-Analysis

The 15 studies included in the present review performed very similar during quality assessment and had comparable, objectively measured outcomes (Newcastle-Ottawa scores 7–8; Table 3). They spanned a period of 10 years and were conducted on patients of institutions from distinct USA states as well as countries from northern and southern Europe. Analysis of the non-LT patients was performed with 12 studies totaling 655 cirrhotic patients with no history of data from OHE and 440 with at least one prior OHE episode. The post-LT analysis included six studies totaling 151 patients with and 156 patients without a history of OHE. For studies evaluating the effect of overt HE after LT, the time after LT varied from 6 months (Acharya et al., 2017) to up to 5 years (Hopp et al., 2019).

**Table 3:** Newcastle-Ottawa scale for risk of bias assessment of the studies included in the meta-analyses.

**Quality assessment criteria**	**Acharya et al. (2017)**	**Bajaj et al. (2011)**	**Bajaj et al. (2013)**	**Garcia-Martinez et al. (2011)**	**Hopp et al. (2019)**	**Nardelli et al. (2017)**	**Riggio et al. (2011)**	**Sotil et al. (2009)**	**Umapathy et al. (2014)**	**Zarantonello et al. (2019)**
**Selection**										
Representativeness of exposed cohort	*	*	*	*	*	*	*	*	*	*
Selection of the non-exposed cohort	*	*	*	*	*	*	*	*	*	*
Ascertainment of exposure	*	*	*	*	*	*	*	*	*	*
Demonstration that outcome of interest was not present at start of study	–	*	–	-	–	-	–	-	–	–
**Comparability**										
Study controls adjusted for main factor?	*	*	*	*	*	*	*	*	*	*
Study controls adjusted for additional factors?	*	–	*	*	*	–	*	*	–	*
**Outcome**										
Assessment ofoutcome	*	*	*	*	*	*	*	*	*	*
Was follow-up longenough for outcometo occur (2 years)	*	*	*	*	*	*	*	*	*	*
Adequacy offollow-up ofcohorts? (80%)	*	*	*	*	*	*	*	*	*	*
Overall Quality Score (Maximum: 9)	**8**	**8**	**8**	**8**	**8**	**7**	**8**	**8**	**7**	**8**

*The studies receiving at least six stars (maximum of nine) were classified as good quality*.

**Figure 2:**
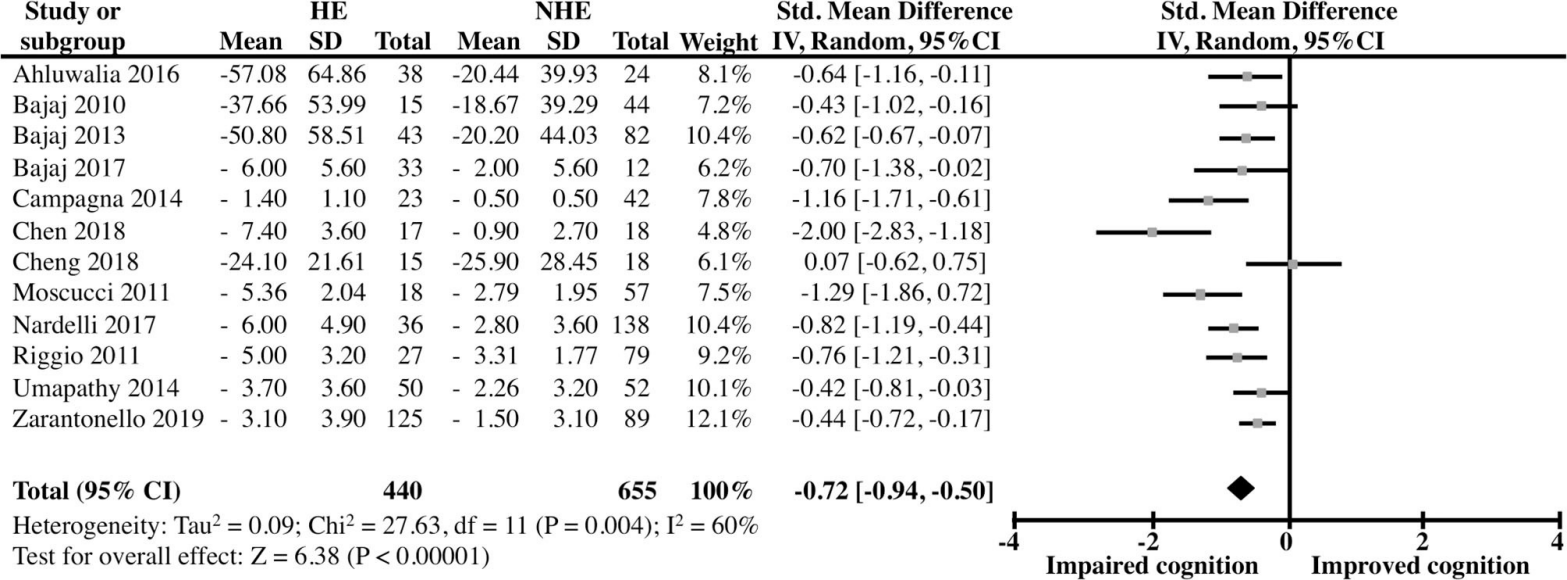
Forest Plot representing the cognitive abilities, as evaluated by several neuropsychological test batteries, of clinically unimpaired cirrhotic patients as a function of HE history.

**Figure 3:**
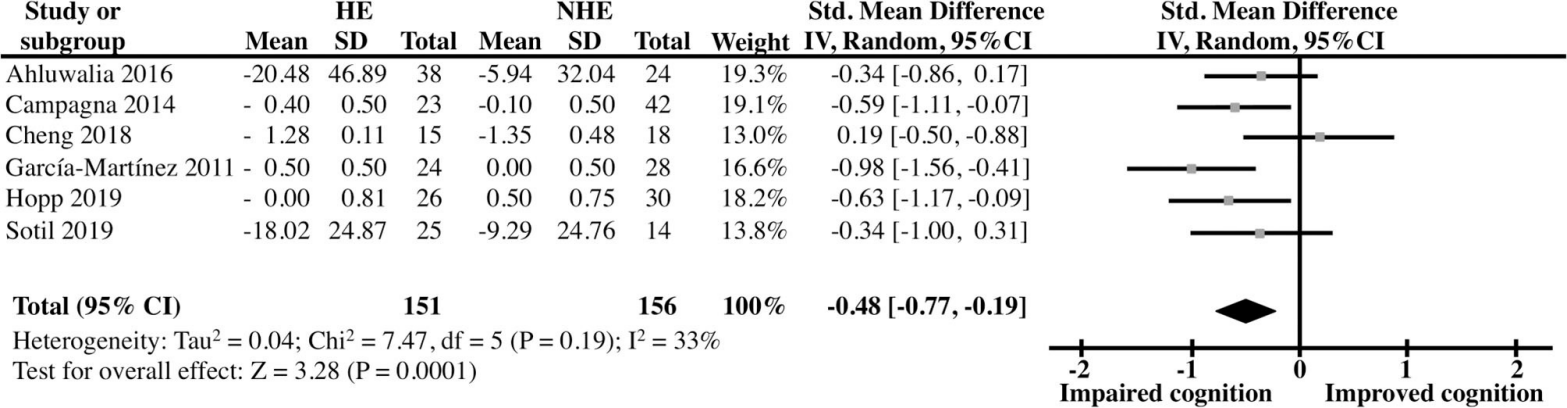
Forest Plot representing the cognitive abilities, as evaluated by several neuropsychological test batteries, of clinically unimpaired cirrhotic patients that have received liver transplantation as a function of HE history.

Moderate heterogeneity was obtained in both the non-LT (I2 = 60%) and the LT (I2 = 33%) analyses which was expected given the continuous nature of the analyzed outcomes (Alba et al., 2016) as well as the considerable clinical variability (i.e., geographic location, etiology of cirrhosis, etc., Table 4 and Supplementary Table 2). In the first analysis, the comparison between the OHE and NHE groups unveiled a robust, highly significant deleterious effect of OHE history on cognitive function in cirrhotic patients, yielding a pooled Std Mean Difference [95% CI] of −0.72 [−0.94 to −0.50] (*Z* = 6.38, *P* < 0.00001, *n* = 12) (Figure 2). On the other hand, the pooled outcomes from the studies examining cognitive function in the same two groups of patients after LT showed a modest but significant difference between the groups suggesting a lingering effect of prior OHE on cognition even after LT [Std Mean Difference (95% CI) = −0.48 (−0.77, −0.19); *Z* = 3.28, *P* < 0.0001, *n* = 6] (Figure 3).

**Table 4:** Characteristics of the studies included in the meta-analyses.

**References**	**No. Healthy controls**	**No. Cirrhotic NHE history patients**	**No. Cirrhotic OHE history patients**	**Type of study**	**Etiology**
Ahluwalia et al. (2016)	0	24	38	PC	HV:23; Alcohol: 6; HV+Alcohol: 10; Others: 23
Bajaj et al. (2011)	0	44	15	CS/P	HV: 41; Alcohol: 9; Others:9
Bajaj et al. (2013)	51	82	43	L	HV: 68; Alcohol: 11; HV+Alcohol: 10; Others: 37
Bajaj et al. (2017)	45	12	33	PC	HV: 21; Alcohol: 10; Others: 14
Campagna et al. (2014)	0	42	23	PC	HV: 38; Alcohol: 13; HV+Alcohol: 9; Others: 5
Chen et al. (2018)	18	18	17	T	HV: 24; Alcohol: 6; HV+Alcohol: 2; Others: 3
Cheng et al. (2018)	30	18	15	PC	HV: 25; Others: 8
Garcia-Martinez et al. (2011)	0	24	28	PC	HV: 25; Alcohol: 16; HV+Alcohol: 8; Others: 3
Hopp et al. (2019)	55	30	26	P sc	HV: 13; Alcohol: 6; Others: 37
Moscucci et al. (2011)	0	57	18	T	HV: 51; Alcohol: 18; Others: 6
Nardelli et al. (2017)	0	138	36	P mc	Not mentioned
Riggio et al. (2011)	0	79	27	CS	Not mentioned
Sotil et al. (2009)	20	25	14	RC	HV: 13; Alcohol: 11; Others: 15
Umapathy et al. (2014)	0	52	50	P/L	Alcohol: 67; Others: 35
Zarantonello et al. (2019)	0	90	124	CS	HV: 73; Alcohol: 64; Others: 77

*CS, Cross-Sectional; HV, Hepatitis Virus (B and/or C); L, Longitudinal; Prospective cohort; P, Prospective; sc, single-center; RC, Retrospective Cohort; T: transversal study*.

## Discussion

Recently, there has been a growing interest in identifying residual cognitive deficits in patients with a history of HE. Our results show that a single episode of HE is sufficient for residual negative cognitive effects to remain, even after the resolution of the episode (Figures 2, 3). Only one of the revised studies (Cheng et al., 2018) reported similar cognitive performance in patients in previous OHE vs. NHE patients. This is probably due to the fact that this study only analyzed Number Connection Test (NCT)-A and Digit Symbol Test (DST), two studies that were previously shown to be less sensitive to a history of OHE (Nardelli et al., 2017).

It seems that learning is the most deteriorated ability in OHE patients; nevertheless, some psychometrical tests have shown sensitivity to detect other cognitive functions that are influenced by OHE episodes. In this sense, Zarantonello et al. (2019) found that MHE patients have poor performance on PHES (PHES score ± SD MHE: −2.1 ± 2.6; Mild OHE: −6.4 ± 3.9) and slower EEGs; alterations that get worse in patients with a history of OHE. Besides, the severity of HE experienced by the patients and the type of tests they performed influence the alterations of learning processes. For instance, regardless the health record or the severity of HE, all patients improve selective attention, visuo-spatial search, cognitive processing speed, and motor speed measure in second evaluation of Trail Making Test (TMT)-A and sRT test; but only patients with mild OHE improved cRT, which implies that OHE is sensitive to decision making function. Furthermore, patients with OHE have a higher MELD score (MELD score ± SD MHE: 13.4 ± 4.8; Mild OHE: 15.7 ± 6.1), a model widely used to predict mortality in patients with cirrhosis (Zarantonello et al., 2019).

Also, our data evidence that, regardless of the etiology, patients with a history of OHE have a higher risk of persistent cognitive impairment (Table 4). However, Umapathy et al. (2014) found that patients with alcoholic cirrhosis also have a higher risk to develop HE (Umapathy et al., 2014).

Pharmacological intervention could ameliorate the condition, but if the condition is severe, LT is the most effective treatment. Our results suggest that while clear improvements are observed after LT, some cognitive impairments still persists in LT patients with a previous episode of HE (Figure 3). Some reports have indicated an improvement in neuronal activity in cortical areas of cirrhotic patients undergoing LT surgery and a slow memory recovery, under evaluation in psychometric test (Senzolo et al., 2009; Ferman et al., 2019). Also, LT restores brain activity of areas involved in motor functions, attention, vision, and working memory as well as psychometric performance in cirrhotic patients with and without a history of OHE. However, in areas like the rectus, brain function was not restored one month after the LT. Furthermore, dysfunction in superior frontal gyrus, lingual gyrus, inferior occipital gyrus, thalamus, putamen, and caudate structures was manifested in cirrhotic patients with previous OHE after LT (Zhang et al., 2017). Moreover, around 30% of cirrhotic patients develop post-transplant encephalopathy, and brain metabolites alterations (glutamine/glutamate increased, myo-Inositol, and choline decreased) have been found in these patients (Pflugrad et al., 2019). Then, recuperation is a slow process, and requires a long-term monitoring in order to detect post-operatory alterations or residual dysfunction.

In the studies analyzed in this work, Cheng et al., 2018 reported that LT produced a significant improvement in NCT-A and DST test scores in both NHE and OHE patients and the study of Ahluwalia et al. (2016) showed that LT improved the score in all the PHES tests in OHE patients. This suggests that the beneficial effects of LT on cognition is not related to the complexity of the tasks involved.

Some evidence has suggested that the presence of MHE can predict the development of OHE, which is characterized by a wide range of symptoms such as fatigue, disorientation, change in personality, bizarre behavior, slurred speech, somnolence, and also, the patient could fall in a coma state (Shiha and Mousa, 2019). Even when some reports indicate that MHE and OHE could be reversible with the proper treatment, the evidence reviewed in here suggests the presence of lingering, cognitive impairment after the complete resolution of these conditions (Figure 2). For instance, a single episode of OHE is accompanied by alteration in the learning response inhibition in the Inhibitory Control Test (ICT), and the severity of the alteration increased with the number of previous OHE episodes experienced. Furthermore, the appearance of new alterations in reaction time, divided attention and working memory basic cognitive domains like psychomotor speed (Bajaj et al., 2011) in OHE patients is common. Similarly, cirrhotic patients, that experience a single or multiple episodes of OHE, have shown learning capacity impairment measured by PHES battery repetition (Riggio et al., 2011; Umapathy et al., 2014). In addition cirrhotic patients with MHE treated with lactulose or Rifaximin showed improvement in PHES one month after the HE episode, but this effect was not evident in patients with previous episodes of OHE, showing persistent cognitive impairment (PHES Day 1: no HE: −3.13 to −1.40, vs. HE: −4.71 to −2.68; PHES Days 30–60: no HE: −1.91 to −0.38 vs. HE: −3.69 to −1.74) (Umapathy et al., 2014). Likewise, cirrhotic patients with prior HE, from a multicenter study, showed difficulties to perform PHES and ICT, in comparison with no-HE cirrhotic patients. These patients showed learning capability loss, despite the therapeutic treatment. In fact, the impairment in cognitive functions after experiencing an episode of OHE is consistent regardless the demographic factor (Nardelli et al., 2017).

The use of several neuropsychological tests has been proposed to detect early cognitive deficiencies before the development of HE. In this context, the Wechsler Memory Scale (WMS) and the Simple Visual Reaction Time (SVRT) tests have shown efficacy in the detection of early alterations of learning, working memory, and visual reproduction in MHE patients infected with hepatitis C virus (HCV). Interestingly, the cognitive alterations observed in HCV patients (especially logic memory, visual reproduction, and memory quotient) were more severe than those caused by other HE etiologies, like cirrhosis (Hashemi et al., 2015). The same observation was reported by Lee and colleagues (2015), who performed the Seoul Neuropsychological Screening Battery, that is a robust battery of neuropsychological tests, to evaluate cognitive deficiencies in viral and alcoholic patients with cirrhosis without OHE evidences. They found decreased memory function, especially in recall and recognition, immediate and delayed recall, semantic, and phonetic aspects. Moreover, alteration in recognition, visuo-spatial, and executive functions were worse in alcoholic than HCV patients, which is consistent with the intensity of the diffused atrophy in the brain showed by alcoholic patients. Hence, it seems that the election of the correct psychometric tests, depending on the etiology, would be a helpful strategy to detect early HE (Lee et al., 2015). Likewise, cirrhosis, alcohol misuse, and HCV infection are etiologies of the expression of differentially cognitive alteration. For example, cirrhosis *per se* deteriorates phonetic verbal fluency and visual attention while patients with chronic alcohol misuse or HCV infection show impairment in working memory. Chronic alcohol misuse patients also have shown deterioration in executive functions. Besides, both factors, cirrhosis and misuse of alcohol, modify the EEG; and the combination of both conditions increases the EEG alterations (Campagna et al., 2014). Finally, there are several good approximations to predict OHE episodes (Riggio et al., 2015; Tapper, 2019; Wernberg et al., 2019). For example, Tapper (2019) published an interesting report in which he highlights the importance of predicting OHE in cirrhotic patients, and reviewed the strategies to predict and diagnose HE using psychometric and neurophysiologic tools, also informing the risks to patients and implementing interventions to mitigate HE progression (Tapper, 2019).

Beyond LT, pharmacological treatment of liver disease can counteract the cognitive impairment observed in cirrhotic patients with and without HE. For instance, interferon therapy has shown efficacy improving cognitive functions of HCV patients with cirrhosis (Barbosa et al., 2017). Moreover, gut-specific antibiotic Rifaximin therapy has shown to improve cognition and working memory performance of MHE patients on N-back task (8 baseline vs. 10 end-of-trial, *p* < 0.05). During this task, the patients showed higher activation on the left parietal operculum and subcortical regions like caudate, thalamus, and hippocampus compared with pre-treatment tests. Also, Rifaximin optimized the ICT, producing a reduction of lures, which is associated with decrease in fronto-parietal regions activation, mediating the inhibitory control network, pointing to a role of Rifaximin in the regulation on the gut-liver-brain axis (Ahluwalia et al., 2014). Furthermore, Acetyl-L-carnitine (ACL) has shown efficacy ameliorating cognitive impairment and biochemical alterations observed in severe HE patients. Apparently, ACL improved attention, learning, psychomotor speed, visuo-constructional function domains, and the capability to remember previously learned information. This improvement was accompanied by a reduction of ammonia levels and modification of EEGs; suggesting that ACL could be used as a therapeutic agent to treat patients with liver damage and HE (Malaguarnera et al., 2011). Other non-pharmacological factors might also ameliorate the cognition deficits triggered by MHE. Vaissman and colleagues have demonstrated that MHE may improve patients' attention and executive functions (measuring by the Mindstreams test) by eating breakfast, since protein-calorie malnutrition is an important aspect of liver diseases (Vaisman et al., 2010).

Another point to discuss is the different tools to diagnose and/or evaluate HE, in all their levels (variants). In this regard, clinical research not only has been focusing on attention, memory, learning, psychomotor, perception, and language functions but also intellectual, functional, emotional, and mood disorders (Prakash and Mullen, 2010; Ferenci, 2017). To detect neurological abnormalities, especially in clinically asymptomatic patients or patients with MHE, specialists resort to adequate neuropsychological test batteries to evaluate cognitive function. Most of the diagnosis tests include a set of psychometric tests to asses cognitive deterioration, which are instruments designed and adapted to different populations around the world, including common tests such as PHES, DST, Block Design Test (BDT), NCT-A and B, the ICT, Auditory Verbal Learning Test (AVLT), Letter and Semantic Fluency tests (LF and SF), TMT-A and B, and Mental Rotation Test (MRT) (Supplementary Table 1). Moreover, other methodologies aimed at evaluating brain function are useful to assess neurophysiological deterioration, these methods include: electroencephalogram (EEG), Magnetic Resonance Spectroscopy (MRS), functional Magnetic Resonance Imaging (fMRI), Diffusion-Tensor Imaging (DTI), Amplitude of Low-Frequency Fluctuation (ALFF), and even the evaluation of some physiological parameters like biological markers (Campagna et al., 2014). Thereby, the selection of the appropriate method(s) is crucial in the precise characterization of the cognitive functions affected, as well as the improvement (if any), after a therapeutic intervention like LT. As mentioned above, MHE is the mild clinical form of HE and is defined as the absence of clinical symptoms and signs, but with some cognitive deficits, evidenced by psychometric tests (Shiha and Mousa, 2019). Hence, one of the psychometric tests often used to diagnose MHE is the PHES; which consists of a battery of five tests: DST, NCT-A, NCT-B, Serial Dotting Test (SDT), and line tracing test (LTT), that evaluate psychomotor skills, concentration, attention, visual perception, visual orientation, visual construction, and memory (Duarte-Rojo et al., 2011). PHES is validated and used in many countries like Germany, Spain, Mexico, and India. Nevertheless, PHES is not always sensitive to detect early neurological alterations in cirrhotic patients, while other psychometric tests like Symbol Digit Modalities (oral SDMT), d2 test, bimanual, and visuomotor coordination test are more sensitive to detect mild neurologic impairment (Gimenez-Garzo et al., 2017). However, some other protocols have been implemented and performed in certain populations, thereby, they need to be validated. In this respect, the use of the NCT, DST, and the Wechsler Memory Scale Chinese revised (WMS-CR) also have shown sensitivity to identify working memory deficits in Chinese cirrhotic patients with and without MHE. In this line, Ciecko-Michalska et al. (2013) also performed MMSE and the following tests: AVLT, Letter and Semantic Fluency Tests (LF and SF), TMT-A and B), Digit Symbol Test (DST), BDT, and MRT to evaluate a wide range of cognitive domains. This protocol includes the evaluation of the number and type of errors made, and differs from the PHES and RBANS tests, that are focused on the psychomotor speed and the efficiency domains. Thus, patients with cirrhosis showed a tendency to make more errors and intrusions [cirrhosis patients: 0.59 (0.81), control group: 0.23 (0.59), *p* < 0.006, FDR-*p* 0.05], apparently because these patients have difficulties to recognize the information during the task, but no other cognitive deficiency was detected (Ciecko-Michalska et al., 2013). Nevertheless, PHES needs to be applied by a qualified specialist; thereby computational tests were developed, like the ICT. This latter test evaluates sustained attention and the ability to inhibit responses to important stimuli during a demanding working memory task. Amodio et al. (2010) performed the ICT immediately after the PHES and noticed that cirrhotic patients have more lures (inhibition inability; 23.2 ± 12.8 vs. 12.9 ± 5.8, *P* < 0.01) and less target accuracy (attention ability; 0.88 ± 0.17 vs. 0.96 ± 0.03, *P* < 0.01) compared with healthy patients. The authors explain that even when ICT provides cognitive information related to the diagnostic standards recommended for MHE investigation, this test “is influenced by demographic variables and exhibits some learning effect,” therefore it needs to be adjusted by target accuracy (Amodio et al., 2010). Similarly, Cona et al. (2014) observed a reduction in the ICT performance during the *detect, go*, and *no-go* trials only in patients with MHE (76 ± 17%), compared with both patients without MHE (86 ± 15%) and healthy controls (90 ± 9%), for all types of trials [*F*_(2, 45)_ = 7.40; *p* < 0.01], which indicates abnormalities in working memory and attention. Moreover, the event-related potentials (ERPs) evaluation indicate that cirrhotic patients with and without MHE have a delay in the latency of P3b (P3b latency: patients with MHE [588(150)] compared with both patients without MHE [544(95)] and healthy controls [529(102)] [*F*_(8, 180)_ = 2.19; *p* < 0.05, g2p = 0.09)] and reduction in P3a amplitude [P3a amplitude: (both with (491(142)] and without MHE [488(164)] than in healthy controls [502(149)] [*F*_(2, 92)_ = 3.44; *p* < 0.05, g2p = 0.07)] in over fronto-temporal regions, suggesting alterations in the attentional processes, even when cirrhotic patients without MHE did not show any abnormalities in psychometric tests. These data denote that the evaluation of attention domains could be the slight manifestation of liver damage, even before the mild manifestation of HE (Cona et al., 2014). In consistence, another study used the ERP and the N-back task was to apply, both report decreasing amplitude of P3 component [*F*_(1.68)_ = 4.72; *p* < 0.05)], which was consistent with the impairment in working memory and executive attention observed in patients with cirrhosis without evident HE. Then, the sensitivity to detect slight changes in neuronal activity of ERP represents an important characteristic to consider as a complementary test to detect MHE (Ciecko-Michalska et al., 2012).

We have to consider the limitations of the present work. First, the reduced number of studies that were included, even when we found 29 articles fitting the inclusion criteria, the characteristics of the analyzed groups allow to include 15 studies in the meta-analyses. This reduced number of studies did not permit us to conduct more specific analysis, separating groups of patients by specific etiologies or treatment history. Also, some studies included in our review did not differentiate between MHE and OHE in their “prior HE” group of patients. Despite this, the heterogeneity of the analyses was moderate: 60 and 30%, for non-LT and LT patients, respectively. This restriction prevents to predict in an OHE population the potential to recover cognition functionality after resolution of the OHE episode or LT.

## Future Directions

HE is one of the most disabling health conditions caused by a complication of liver disease, which lead to a wide spectrum of neuropsychiatric manifestations and cognitive complications including memory (verbal, visual, working), learning, attention, language, perception, psychomotor, and intellectual functions. Cognitive impairments differ in severity; while some patients do not experience overt clinical symptoms, others show serious complications sometimes leading to coma. Several neuropsychological tests and batteries have been developed and used to evaluate cognitive impairment of HE patients. In turn, the tests differ in specificity and sensitivity. Other factors also influence the gravity of the impairment such as the degree of liver damage, early cirrhosis, HCV infection, and the existence of a previous HE episode. To our knowledge, this is the first meta-analysis exploring the long-term effect of a resolved HE episode on cognition and whether LT does not restore completely the cognitive capabilities. However, the mechanisms underlying these results have not been explored yet. As liver disease rates have been steadily increasing over the years, the validation of all these neuropsychological tests and batteries in other populations will be necessary, considering that chronic liver disease occurs throughout the world irrespective of sex, geographic region, or ethnicity. In addition, further neurophysiological studies on different populations of HE patients should help deepen our understanding of its functional underpinning, perhaps guiding the way through the design of novel treatments and prevention strategies.

## Data Availability Statement

The raw data supporting the conclusions of this article will be made available by the authors, without undue reservation.

## Author Contributions

RZ, GR-R, and J-PM: conceptualization. CJ-P, AC-S, and TM-J: methodology. MF-M and OL-F: validation. J-PM, CJ-P, AC-S, and TM-J: formal analysis. CJ-P, AC-S, TM-J, and DID: investigation. RZ and CJ-P: resources. JP-M, AC-S, TM-J, and OL-F: data curation. RZ, OL-F, CJ-P, AC-S, and TM-J: writing—original draft preparation. CJ-P, RZ, J-PM, and GR-R: writing—review and editing. CJ-P, TM-J, and AC-S: visualization. RZ: supervision. RZ, J-PM, AC-S, TM-J, and CJ-P: project administration. J-PM, GR-R, OL-F, and RZ: funding acquisition. All authors have read and agreed to the published version of the manuscript.

## Conflict of Interest

The authors declare that the research was conducted in the absence of any commercial or financial relationships that could be construed as a potential conflict of interest.
