# Corrigendum: Cognitive Impairment After Resolution of Hepatic Encephalopathy: A Systematic Review and Meta-Analysis

**DOI:** 10.3389/fnins.2021.694239

**Published:** 2021-05-28

**Authors:** Óscar López-Franco, Jean-Pascal Morin, Albertina Cortés-Sol, Tania Molina-Jiménez, Diana I. Del Moral, Mónica Flores-Muñoz, Gabriel Roldán-Roldán, Claudia Juárez-Portilla, Rossana C. Zepeda

**Affiliations:** ^1^Laboratorio de Medicina Traslacional, Instituto de Ciencias de la Salud, Universidad Veracruzana, Xalapa, Mexico; ^2^Laboratorio de Neurobiología de la Conducta, Departamento de Fisiología, Facultad de Medicina, Universidad Nacional Autónoma de Mexico, Ciudad de Mexico, Mexico; ^3^Facultad de Biología-Xalapa, Universidad Veracruzana, Xalapa, Mexico; ^4^Instituto Interdisciplinario de Investigaciones de la Universidad de Xalapa, Xalapa, Mexico; ^5^Programa de Doctorado en Ciencias Biomédicas, Universidad Veracruzana, Xalapa, Mexico; ^6^Laboratorio de Biomedicina Integral y Salud, Centro de Investigaciones Biomédicas, Universidad Veracruzana, Xalapa, Mexico

**Keywords:** learning, cognition, liver disease, cirrhosis, impairment, psychometric test, liver transplant

In the original article, there was an error in the value of Std. Mean Difference stated in the last sentence of the Abstract.

A correction has been made to *Abstract* section. The corrected Abstract is shown below.

Hepatic encephalopathy (HE) is one of the most disabling metabolic diseases. It consists of a complication of liver disease through the action of neurotoxins, such as excessive production of ammonia from liver, resulting in impaired brain function. Its prevalence and incidence are not well known, although it has been established that up to 40% of cirrhotic patients may develop HE. Patients with HE episodes display a wide range of neurological disturbances, from subclinical alterations to coma. Recent evidence suggests that the resolution of hepatic encephalopathy does not fully restore cognitive functioning in cirrhotic patients. Therefore, the aim of this review was to evaluate the evidence supporting the presence of lingering cognitive deficits in patients with a history of HE compared to patients without HE history and how liver transplant affects such outcome in these patients. We performed two distinct meta-analysis of continuous outcomes. In both cases the results were pooled using random-effects models. Our results indicate that cirrhotic patients with a history of HE show clear cognitive deficits compared to control cirrhotic patients (Std. Mean Difference (in SDs) = −0.72 [CI 95%: −0.94, −0.50]) and that these differences are not fully restored after liver transplant (Std. Mean Difference (in SDs) = −0.48 [CI 95%: −0.77, −0.19]).

The authors apologize for this error and state that this does not change the scientific conclusions of the article in any way. The original article has been updated.

