# Supplementary material for: Cognitive Impairment After Resolution of Hepatic Encephalopathy: A Systematic Review and Meta-Analysis
Source: Front Neurosci. 2021 Mar 10;15:579263. doi: 10.3389/fnins.2021.579263 (PMC8006450; doi:10.3389/fnins.2021.579263)
Supplement: Supplementary file 1 [file Table_1.docx]

Supplementary Table S1. Description of the studies included in the analytical review.

| First author and year | Type of  study | Country | Gender | Mean age  (Years) | Goal | Result |
| --- | --- | --- | --- | --- | --- | --- |
| Acharya 2017 | Mixt | USA | M | 61 - 64 | To define learning/test-sophistication on EncephalApp (downloadable Application) in OHE patients compared to no-OHE patients and controls cross-sectionally and longitudinally. | 89 controls and 230 cirrhotics (85 prior OHE). Cirrhotic patients had impaired EncephalApp total times and impaired learning on the On runs compared to controls. OHE patients had worse EncephalApp times and learning with the On runs compared to no-OHE patients, which persisted in the longitudinal cohort. No differences in learning were seen in the Off runs. After transplant there was restoration of learning capability with the On runs in the OHE patients. |
| Ahluwalia 2014 | L | USA | F/M | 60 | To evaluate the effect of rifaximin on the gut-liver-brain axis in MHE. | Pre-rifaximin patients had cognitive impairment. At trial-end, there was a significantly higher correct 2-back responses, ICT lure inhibitions and reduced endotoxemia (p= 0.002). Along with a significantly higher activation from baseline in subcortical structures (thalamus, caudate, insula and hippocampus) and left parietal operculum during N-back, decrease in fronto-parietal activation required for inhibiting lures, including left parietal operculum during ICT. |
| Ahluwalia 2016 | P | USA | Not mentioned | 21-65 | To evaluate the neurometabolic and functional brain changes as modulators of cognition and quality of life after transplant in patients with cirrhosis who were with/without pretransplant cognitive impairment and HE. | Quality of life was significantly worse in CI and HE groups before transplant. After transplant, there was (1) significantly lower brain activation needed for lure inhibition; (2) reversal of pretransplant ammonia-associated changes; and (3) improved white matter integrity. |
| Bajaj 2010 | Mixt | USA | F/M | 50 - 56 | To explore the extent of residual cognitive impairment following clinical resolution of OHE. | In study A, 54 had experienced OHE, 120 had MHE and 52 with no MHE. Despite normal mental status on lactulose after OHE, cirrhotics were cognitively impaired, based on results from all tests. Learning of response inhibition (ΔL1–2 ≥1), was evident in patients with MHE and no MHE, but was lost after OHE. In study B, the number of OHE hospitalizations correlated with severity of residual impairment, indicated by ICT lures (r=0.5, P=0.0001), DST (r=−0.39, P=0.002) and NCT-B (r=0.33, P=0.04). In the prospective study, 15 developed OHE; ICT lure response worsened significantly after OHE (12 before vs.18 after, P=0.0003) and learning of response inhibition was lost. The 44 patients who did not experience OHE did not have deteriorations in cognitive function in serial testing. |
| Bajaj 2013 | Mixt | USA | F/M | 50± 7 | To test the validity of the Stroop Smartphone App as a screening tool for MHE. | The App showed a 78% sensitivity and a 90% specificity; worst outcome in cirrhotic patients with previous OHE history. For established MHE diagnosis tools, scores for patients with previous OHE *vs* patients without previous OHE were as follows: ICT lures: 14±8 vs. 10 ±8; %ICT targets: 89±18 vs. 96±7; ICT random: 14±15 vs. 8±4; WLs: 23±18 vs. 12±12; MHE using PHES >4SDs: 79% vs. 54%. All with p<0.0001. |
| Bajaj 2017 | L | USA | M | 21-65 | To determine the effect of LT on gut microbiota and to determine whether gut microbiota are associated with cognitive dysfunction after LT. | HRQOL significantly improved, PHES, with increase in microbial diversity, increase in autochthonous, and decrease in potentially pathogenic taxa were seen after LT compared with baseline. However, there was continued dysbiosis and HRQOL/cognitive impairment after LT compared with controls in 29% who did not improve PHES after LT. In these, Proteobacteria relative abundance was significantly higher and Firmicutes were lower after LT, whereas the reverse occurred in the group that improved. Delta PHES was negatively correlated with delta Proteobacteria and positively with delta Firmicutes. |
| Campagna 2014 | R | Italy | F/M | 52± 9 | To assess the influence of alcohol misuse, HCV infection and cirrhosis per se on the neuropsychological and EEG profile and to evaluate the role of alcohol misuse and HCV infections as potential confounding factors in the detection of MHE. | Cirrhosis, chronic alcohol misuse and HCV infection are associated with cognitive dysfunction. The interaction with alcohol misuse in cirrhotic patients further impact on brain dysfunction. Cirrhosis was associated with low PVF and difference between TMT B-A. Chronic alcohol misuse was associated with low PVF, TMT (B-A), ITM 10 and 30 s. The EEG alterations were pronounced in patients with alcoholic cirrhosis. |
| Chen 2018 | CS | China | F/M | 51± 9 | To investigate structural brain connectome alterations in cirrhotic patients with prior OHE. | A progressive decrease trend for network strength, global efficiency and local efficiency was found from non-prior- OHE to prior-OHE, compared with HC. Among the three groups, the regions with altered nodal efficiency were mainly distributed in the frontal and occipital cortices, paralimbic system and subcortical regions. The topological metrics, such as network strength and global efficiency, were correlated with PHES among cirrhotic patients. |
| Cheng 2018 | P | China | F/M | 53± 8 | To investigate the impact of prior HE episodes on post-transplantation brain function recovery. | Long- and short-range FCS analysis indicated that before transplantation both noHE and HE groups showed diffuse FCS abnormalities relative to healthy controls. For the noHE group, the abnormal FCS found before LT largely returned to normal levels after LT, except for in the cerebellum, precuneus, and orbital middle frontal gyrus. However, the abnormal FCS prior to LT was largely preserved in the HE group, including high-level cognition-related and vision-related areas. |
| Ciecko-Michalska 2012 | P | Poland | F/M | 30 - 50 | To examine brain activity and cognitive functioning in patients with liver cirrhosis without MHE or overt clinical HE. | Effects of decreased performance as well as a decrease in P3 amplitude with difficulty level were identified. N-back task is very sensitive in detection of subtle neurocognitive alterations in liver cirrhosis. Patients whose neuropsychological performance is within the normal range can still reveal subtle changes in CNS functioning visible, confirming the usability of this method in diagnosis of neurocognitive functions in patients with liver cirrhosis. |
| Ciecko-Michalska 2013 | P | Poland | M | 40.91 ± 10.87 | To provide a profile of cognitive functioning in patients with liver cirrhosis, through the assessment that covers a wider range of cognitive functions than the usual screening battery. | There is no specific profile of cognitive disturbances in MHE, as patients with liver cirrhosis commit significantly more errors of intrusions in the AVLT during the delayed free recall trial. Results significantly deviating from the norm in at least 2 tests were found only in 7 cirrhosis patients. |
| Ferman 2019 | L | USA | M | ≤60 and >60 | To determine whether age or clinical morbidities were associated with pre- and post-transplant executive and memory performance using the BTACT. | Older recipients were more likely to have hepatocellular carcinoma, a lower biologi- cal MELD score at transplant, less cellular rejection, and fewer post-operative hospital days. Older and younger recipients showed comparable pre-transplant executive and memory function and comparable post-transplant improvement. Both older and younger patients showed statistically significant improvement in executive function scores at 3 months post-transplant and maintained improvement at 6 and 9 months. Memory function improved significantly in older patients by 6 months post-transplant but did not improve significantly in the younger group. |
| García-Martínez 2011 | P | Spain | F/M | 44 - 64 | This study analyzes the relationship of HE with cognitive function and brain volume after transplantation. | All cognitive indexes improved after liver transplantation, but 7 patients (13%) showed persistent mild cognitive impairment. Global cognitive function after transplantation was poorer in patients with the following variables before liver transplantation: alcohol etiology, diabetes mellitus, and HE. Brain volume after transplantation was smaller in patients with prior HE. |
| Hopp 2019 | P | Germany | F/M | 38 - 66 | To determine whether a history of HE before liver transplant had an impact on the long-term outcome of cognitive function after liver transpalnt and if patients who underwent liver transpalnt 5 years earlier showed worse cognitive function than healthy controls. | Before liver transplant, patients performed significantly worse than controls in the psychometric tests: RBANS Total Scale (TS), 92.6 ± 13.3 vs 99.9 ± 12.0, P = 0.01; and PHES, 0 (−3 to 1) vs 1 (0-2), P < 0.001. At 1 year after liver transplant, patients with a history of HE still showed cognitive impairment compared with controls: RBANS TS, 89.8 ± 15.1 versus 99.9 ± 12.0, P < 0.01; and PHES, 0 (−2 to 1.25) vs 1 (0-2), P = 0.03. At 5 years after liver transplant, patients with and without a history of HE showed normal cognitive function and improved HRQOL. |
| Lauridsen 2020 | CS | Denmark | M | 40-79 | To validate the SIPCHE score for MHE identification in a Danish cirrhosis cohort, using the CRT test and the PHES for MHE diagnosis. The patients were followed for episodes of OHE, and the prediction by SIPCHE was calculated. | SIPCHE score positivity had high sensitivity (82%) but low specificity (38%) for MHE detection. Patients with an abnormal SIPCHE had a higher incidence of OHE during follow-up (35% vs. 14%, *P* = 0.05). OHE prediction sensitivity was 87% and exclusion sensitivity was 85%. The patients with an abnormal SIPCHE had twice as many subsequent episodes of OHE, and despite their high mortality, also a higher risk. An abnormal SIPCHE had a high sensitivity and low specificity for MHE identification. An abnormal SIPCHE was associated with a more than doubled risk of OHE, even with death as a competing event. |
| Lee 2015 | P | South Korea | F/M | 40 - 60 | To determine the differences of cognitive function between viral and alcoholic compensated LC. | The memory function by SVLT and RCFT recognition scores was more significantly impaired in alcoholic compensated LC patients than in those with viral compensated LC. The COWAT supermarket, which was related with frontal/executive function, was more impaired in alcoholic than in viral LC patients. K-MMSE total scores revealed that brain change in the frontal lobe was more dominant in the alcoholic LC group than in the viral LC group (27.5 ± 1.9 and 26.2 ± 3.1, p = 0.03). Patients with compensated LC had decreased memory and frontal functions as revealed by the SVLT (recall and recognition; recognition: 20.1 ± 3.6 and 17.8 ± 4.8, p = 0.022), RCFT (immediate and delayed recall), and COWAT (semantic: semantic: 17.1 ± 6.9 and 12.7 ± 6.9, p = 0.004, and phonemic) as compared to the age education matched normal value. The 1-, 2-, and 3-year cumulative incidence rates of OHE were 23%, 26%, and26% and 33%, 43%, and 49%intheviraland alcoholic compensated LC group, respectively (p = 0.033). |
| Lin 2014 a | L | Taiwan | F/M | 53±10 | To determine (1) whether MHE patients show deterioration of WM integrity after LT by assessing multiple DTI indices (2) whether changes in WM integrity either affected by baseline or interval changes in liver functions after LT and (3) whether there is any correlation between WM integrity and cognitive performance before and after LT. | Transplantation improved extracellular cerebral edema and the results of associated cognition tests. LT significantly corrected liver function in MHE patients, including prothrombin time (p<0.001). After LT, the MHE patients presented with poorer executive function in the abstraction test (p=0.001) compared to healthy subjects. Longitudinal comparison after LT showed significant improvements in executive function (p = 0.006), and visuospatial function (p = 0.007). Comparison between Post-LT and controls showed no significant differences in WM integrity. |
| Lin 2014 b | P | Taiwan | F/M | 26-61 | (1) To show a randomization network topological pattern preLT and postLT by implementing a node-based analytical approach that examined crosscorrelations between regions in five major resting-state networks (DMN, DAN, ECN, SN, and PN) and (2) to test the causality relationships among those major networks during recovery of liver function. | After transplantation, cognitive functions improved with increased functional connectivity. The interaction metrics among large-scale networks in patients became similar to healthy controls. The increase in PN affected the decrease in SN, while the increase in DAN forced a decrease in DMN. There was a bidirectional balance between DMN and SN. Dynamic disruptions and reconstruction in intrinsic large-scale networks are associated with parallel patterns of cognitive information processing deficits and recovery. |
| Malaguarnera 2011 | L | Italy | F/M | 37-64 | To investigate the effects of ALC treatment on cognitive functions in patients with severe HE. | Patients with severe HE treated with ALC showed a decrease of cognitive deficits and an improvement in the domains of attention, learning, psychomotor speed, visuoconstructional skills and the ability to remember previously learned information. 88% of patients treated with ALC vs 72% of patients treated with placebo showed a significant improvement in EEG. The improvement of cognitive deficits, the reduction of ammonia, and the modification of EEG in patients treated with ALC suggest that ALC could represent a new tool in the treatment of severe hepatic encephalopathy. |
| Moscucci 2011 | P | Italy | F/M | 65.3±10.9 | To investigate the relative impact of previous HE and minimal HE in a group of consecutively hospitalized cirrhotic patients to clarify the independent role of each one on HRQoL. | Minimal HE was signifi- cantly more frequent in patients with previous HE than in those without (p < 0.001), independently on the method used for the diagnosis (PHES or SPHES). A deeper impairment in several domains of SF-36 was observed in patients with previous bouts of overt HE, in those with ascites, as well as in those with decompensated cirrhosis. At multivariate analysis, ascites, MELD score and previous HE were independently related to the mental-component-summary (MCS) of SF-36, whereas ascites was the only variable independently associated with the physical-component-summary (PCS) of SF-36. Minimal HE (independently on the method used for its diagnosis) impaired only one domain of SF-36. |
| Nardelli 2017 | P | Italy | Not mentioned | Not mentioned | To evaluate persistence of learning impairment in prior HE in a multicenter study. | Demographics and cirrhosis severity characteristics were similar between sites apart from lower education and higher age in Italian patients. Our study design determined F/M the intra-visit (1st half ICT vs. 2nd half ICT) and inter-visit learning (total ICT and PHES scores) differences between patients with or without prior HE. The within-visit results confirm the prior single-center study, in that patients with prior HE loses their learning capability for ICT lures compared to those without HE. HE subjects had difficulty in improving performance on tests apart from the NCT-A and DST. This translated into a similar performance on the other PHES sub-tests, the total PHES SD score and MHE rate, and the ICT lures and targets. |
| Riggio 2011 | L | Italy | M | 58 ± 11 | To explore how specific cognitive domains predict QoL among liver transplant candidates by replicating Stewart and colleagues’ (2010) 3-factor model of cognitive functioning, and determining how variability in these cognitive domains predicts mental health and physical QoL. | The loss of learning capacity was observed in the patients with previous HE only but was absent in the group of patients without previous HE. The loss of learning capacity seems to be more sensitive than the static evaluation of the patients’ psychometric performance to detect the persistence of cognitive impairment after the resolution of OHE. |
| Riggio 2015 | PO | Italy | F/M | 63 ± 12 | To identify patients with cirrhosis at risk for overt HE. | 68 patients (32%) developed at least 1 episode of overt HE. Based on multivariate analysis, the development of overt HE was associated with previous HE, minimal HE (based on PHES), and level of albumin less than 3.5 g/dL (area under the curve, 0.74). A model that excluded minimal HE but included albumin level and previous HE also identified patients who would develop overt HE (AUC, 0.71); this difference in AUC values was not statistically significant (P = 0.104). Both models were validated in the independent group of patients (3 variables: AUC, 0.74; 95% confidence interval, 0.66–0.83; and 2 variables: AUC, 0.71; 95 % confidence interval, 0.63–0.78). |
| Sotil 2009 | R | Spain | Not mentioned | 57 ± 8 | To compare the cognitive functions in patients that had undergone OLT, depending on whether they had suffered one or more episode of overt HE. | 25 OLT patients with a history of overt HE and 14 OLT patients with no history of HE. Neurocognitive abnormalities were more severe in liver transplant recipients that had suffered from overt HE prior to OLT. |
| Tryc 2014 | Mixt | Germany | F/M | 43-59 | To investigate the course of cognitive function after OLT with consideration of a history of MHE or overt HE, comorbidities, and medications after OLT. | Twelve months after OLT, cognitive dysfunction characteristic of HE had resolved, but a secondary cognitive decline became apparent and had features different from those known with HE. Approximately 70% of the patients deteriorated in at least 1 cognitive domain of RBANS. This cognitive decline was related to neither a history of HE nor a history of alcohol abuse, but it was accompa- nied by a decline in the quality of life. |
| Umapathy 2014 | Mixt | India | Not mentioned | 40 - 47 | To study persistent cognitive impairment in the form of loss of learning capacity in cirrhotic patients, with a previous episode of OHE, despite lactulose/rifaximin therapy. | The loss of learning ability in cirrhotic patients after a previous episode of OHE despite normal mental status. Patients with a previous episode of OHE might be prioritized for liver transplantation because of persistence of cognitive impairment and a higher incidence of posttransplantation neurological complications. |
| Wernberg 2019 | CS | Denmark | M | 40-79 | To examine the single and combined predictive value of the continuous reaction time test and the portosystemic encephalopathy syndrome test for OHE occurrence. | At baseline, the CRT test was abnormal in 74 patients and the PSE in 47. During follow-up 35 patients (27%) experienced 74 OHE events. 23 patients with abnormal CRT experienced OHE (prediction sensitivity 65%). The PSE predicted OHE in 14 patients (prediction sensitivity 40%). One or both tests were abnormal in 87/130 (67%) and this predicted OHE in 27 patients (21%) (prediction sensitivity 77%). |
| Zarantonello 2019 | R | Italy | M | 49 - 69 | To compare performance on the first/second attempt at a series of tests in cirrhotic patients. | Learning was influenced by test type. All patients were capable of improving in TMT-A and sRT, regardless of their history and their HE status. By contrast, only patients with mild OHE on the day of study improved in cRT. |
| Zhang 2017 | L | China | F/M | 52 ± 9 | To investigate alterations of resting-state brain activity before and after LT. | Neuropsychological analyses indicated significant improvement of cognitive function in both groups. ALFF analysis showed that the brain activity in regions regulating motor function, vision, attention, and working memory were restored in both groups, reflecting the neuroplasticity of the brain. However, some persistent impairments and new-onset impairments in other regions related to these cognitive functions were observed in each group. Between-group comparison showed that although cognitive performance improved in both groups, the specific neural basis of LT in each group was different. The significant correlations of altered brain activity in regions showing LT and group effect with altered performance in neuropsychological and biochemical tests suggest a possible neuroimaging marker for the monitoring of short-term recovery of HE and the difference in individual recovery of cognitive performance. |
| Abbreviations: ALC, Acetyl-L-carnitine; ALFF, Amplitude of low-frequency fluctuation; AVLT, auditory verbal learning test; BTACT, Brief Test of Adult Cognition by Telephone; CS, cross-study cohort; MHE, minimal hepatic encephalopathy; CHO, choline; CI, cognitive impairment; CNS, central nervous system; COWAT, controlled oral Ward Association Test; CRT, continuous reaction time; DAN, dorsal attention; DMN, default mode; DST, Digit symbol test; ECN, executive control; EEG, electroencephalogram; FCS, functional connectivity strength; F, female; Glx, glutamine/glutamate; HC, healthy controls; HCV, hepatitis C virus; HE, hepatic encephalopathy; HRQOL, health-related quality of life; ICT, inhibitory control test; ITM, interference task memory; L, longitudinal- study cohort; LC, liver cirrhosis; LDST, Letter Digit Substitution Test; LO, late onset; LT, Liver transplantation; M, male; MCS, mental-component-summary; MELD, model for end-stage liver disease; MHE, minimal hepatic encephalopathy; mI, myoinositol; MMSE, mini-mental state examination; MRS, magnetic resonance spectroscopy; NCT-A, number Connection Test-A; NCT-B, number Connection Test-B; OHE, overt hepatic encephalopathy; OLT, orthotopic liver transplantation; P, prospective; PHES, sychometric Hepatic Encephalopathy Score; PN, primary networks; PO, Prospective observational; PSE, portosystemic encephalopathy; PVF, phonemic verbal fluency; QoL, quality of life; RBANS, Repeatable Battery for the Assessment of Neuropsychological Status; RCFT, Rey-Osterrieth Complex Figure Test; R, retrospective; sRT, simple reaction times; SIPCHE, sick- ness impact profile questionnaire for covert hepatic encephalopathy; SN, salience; SPHES, simplified Psycho- metric Hepatic Encephalopathy Score; SVLT, Seoul-verbal learning test; TMT A/B, trail making test A and B; WM, brain’s white matter. | | | | | | |
