# Supplementary material for: Cognitive Impairment After Resolution of Hepatic Encephalopathy: A Systematic Review and Meta-Analysis
Source: Front Neurosci. 2021 Mar 10;15:579263. doi: 10.3389/fnins.2021.579263 (PMC8006450; doi:10.3389/fnins.2021.579263)
Supplement: Supplementary file 2 [file Table_2.docx]

Supplementary Table 2. Psychometric Tests used to diagnose HE.

| Test | Parameters evaluated | References |
| --- | --- | --- |
|  | **Attention and memory** |  |
| AVL | Is frequently used in neuropsychology literature to comprehensively assess the memory. The test measures verbal learning as immediate and delayed free recall, recognition, and retroactive and proactive interference. | García-Martínez et al., 2011; Ferman et al., 2019. |
| CRT | Is a 10-minute computerized test assessing the patient’s alertness, psychomotor speed, and response inhibition. | Wernberg et al., 2019; Lauridsen et al., 2020. |
| DST | The test measures attention and working memory. It is a key tool for working, verbal and short-term memory. | Campagna et al., 2014; Lee et al., 2015; Zhang et al., 2017; Cheng et al., 2018. |
| SDMT | It measures cognitive functioning, processing speed, working memory, visuospatial processing and attention. | Bajaj et al., 2013. |
| SDMT Oral | It measures cognitive functioning, processing speed, working memory, visuospatial processing and attention. | García-Martínez et al., 2011. |
| ST | Neuropsychometric test examining word-conflict color. Working memory, executive functions, and attention. | Acharya et al., 2017. |
| SVLT | Brief test of verbal learning and memory, which is used to examine dementia patients when a more comprehensive memory assessment is not feasible, or when serial testing is desired. | Lee et al., 2015. |
|  | **Dementia** |  |
| K-MMSE | Is widely used to test cognitive function among the elderly for screening dementia; it includes tests of orientation, attention, memory, language and visual-spatial skills. | Lee et al., 2015. |
| PRT | To assess logical memory, measures of episodic prose recall, outperforms other cognitive and biomarker predictors of cognitive decline to dementia. | Malaguarnera et al., 2011. |
| RBANS | To Identify and characterize abnormal cognitive decline in the older adult, and as a neuropsychological screening battery for younger patients. | Sotil et al., 2009; Tryc et al., 2014. |
| SDT | To assess impaired intellectual capacity (cognitive function). Clinical evaluation of cognitive capabilities, especially in the elderly, in order to assess the presence of dementia and to estimate its extent. | Campagna et al., 2014. |
| WAIS-III | To measure individual's intelligence quotient (IQ), and cognitive ability in adults and older adolescents. Verbal IQ is calculated based on the sum of the following subtests: Vocabulary, Similarities, Arithmetic, Digit Span, Information and Comprehension. Performance IQ is calculated from the sum of the following subtests: Picture Completion, Digit Symbol coding, Block Design, Matrix Reasoning and Picture Arrangement. However, factor analytic studies have suggested that the subtests do not fall neatly into verbal and performance IQ; rather, four factors emerge: Verbal Comprehension, Perceptual Organization, Working Memory and Processing Speed. With the addition of three further subtests (Symbol Search, Letter-Number Sequencing and Object Assembly) it is also possible to calculate scores on these four indices. | Lin et al., 2014a; Lin et al., 2014b. |
|  | **Emotion, depression, and mental disorders** |  |
| BDI | Clinical, neurocognitive and mood assessments, it measures characteristic attitudes and symptoms of depression. | Ahluwalia et al., 2016. |
| ICT | Attention and response inhibition that has been used to characterize attention deficit disorder, schizophrenia, and traumatic brain injury. | Bajaj et al., 2010; Bajaj et al., 2013; Ahluwalia et al., 2014; Tryc et al., 2014; Ahluwalia et al., 2016; Acharya et al., 2017; Nardelli et al., 2017. |
|  | **Language disorders** |  |
| BNT | Confrontational word retrieval in individuals with aphasia or other language disturbance caused by stroke, Alzheimer's disease, or other dementing disorder. | Lee et al., 2015. |
| COWAT | To evaluate frontal lobe executive function through verbal fluency tests, and in particular initial letter fluency. | Lee et al., 2015; García-Martínez et al., 2011; Malaguarnera et al., 2011. |
| K-BNT | To assess language and related function. Most of the items had to be altered due to the linguistic and cultural differences between Korean and English speakers. | Lee et al., 2015. |
|  | **Psychomotor disorders** |  |
| NCT-A | Reaction time and psychomotor speed. | Bajaj et al., 2010; Bajaj et al., 2013; Zhang et al., 2017; Cheng et al., 2018. |
| NCT-B | Psychomotor speed, set shifting, divided attention. | Bajaj et al., 2010; Bajaj et al., 2013. |
|  | **Visuospatial disorders** |  |
| BDT | Is often thought of as a prototype test for visuospatial ability. The results from this test show worse performance in older individuals. It uses block design and a clock test to study visuospatial functioning and spatial orientation. Subjects replicate standarized designs with given blocks in a timed manner. | Bajaj et al., 2013. |
| JLO | Visuospatial perception, visuospatial function. | García-Martínez et al., 2011; Malaguarnera et al., 2011. |
| RCFT | Visuospatial recall memory, visuospatial recognition memory, response bias, processing speed, visuospatial constructional ability. | Lee et al., 2015. |
| VOT | The Hooper VOT is particularly sensitive to neurological impairment. Is a screening instrument that measures the ability to organize visual stimuli. | García-Martínez et al., 2011. |
|  | **Hepatic encephalopathy** |  |
| EncephalApp | Is a test of mental speed for the diagnosis of MHE and the prediction of OHE development. The use of this convenient App may improve the screening process and subsequent treatment rates in potential patients with MHE, especially in the US where the testing is not routinely performed. Abnormalities in attention and psychomotor speed are the hallmark of cognitive impairment in MHE and negatively impact on patient's quality of life. | Bajaj et al., 2013; Acharya et al., 2017; Tapper, 2019. |
| PHES | It is currently considered the diagnostic method of choice for MHE. This battery measures psychomotor speed and precision, visual perception, visuospatial orientation, visual construction, concentration, attention and memory. It is composed by five tests: DST, NCT-A, NCT-B, SDT, and LTT. | Sotil, 2009; Moscucci et al., 2011; Riggio et al., 2011; Tryc et al., 2014; Umapathy et al., 2014; Riggio et al., 2015; Ahluwalia et al., 2016; Acharya et al., 2017; Bajaj et al., 2017; Nardelli et al., 2017; Chen et al., 2018; Hopp, 2019; Tapper, 2019; Wernberg et al., 2019; Zarantonello, 2019; Lauridsen et al., 2020. |
| SIPCHE | It is a diagnostic tool for MHE in patients with liver cirrhosis, in whom MHE diagnosis was given by impairment in two or more of the following psychometric tests: the number connection test A and B, the digit symbol test, and the block design test. | Lauridsen et al., 2020 |
|  | **Other batteries of tests** |  |
| BTACT | Assessement of multiple central dimensions such as episodic memory, working memory, reasoning, verbal fluency, and executive function for effective functioning in adulthood. Is the first instrument, which includes measurements of processing speed, reaction time, and task switching/inhibitory control for use over the telephone. | Ferman et al., 2019 |
| CASI | Provides quantitative assessment on attention, concentration, orientation, short-term memory, long-term memory, language abilities, visual construction, list-generating fluency, abstraction, and judgment. | Lin et al., 2014a; Lin et al., 2014b. |
| HRQOL | It focuses on the impact of health on a person’s ability to live a fulfilling life. HRQOL represents a broad concept of physical, psychological with social functioning and well-being that includes both positive and negative aspects. | Moscucci et al., 2011; Tryc et al., 2014; Ahluwalia et al., 2016; Bajaj et al., 2017. |
| MMSE | Mini-Mental State Examination is compose by AVLT, LF and SF, TMT A y B Trail Making Test, DST Digit Symbol Test, BDT Block Design Test, MRT Mental Rotation Test. | Malaguarnera et al., 2011; Ciecko-Michalska et al., 2013; Ahluwalia et al., 2016; Bajaj et al., 2017; Lauridsen et al., 2020. |
| PROMIS | A set of person-centered measures that evaluates and monitors physical, mental, and social health in adults and children. It can be used in the general population as well as individuals living under chronic conditions. | Ahluwalia et al., 2016 |
| SNSB | It is a battery of six tests: Attention (Digit Span Test), Language and related function (Korean-Boston Naming Test), Visuospatial function (Korea-Mini Mental State Examination), Memory function (K-MMSE): registration and recall, Seoul-Verbal Learning Test (SVLT): immediate and delayed recall/recognition), frontal lobe executive function (Controlled Oral Word Association Test). | Lee et al., 2015. |
| TMT A, B | Provides information on visual search, scanning, speed of processing, mental flexibility, and executive functions.  -TMT-A requires an individual to draw lines sequentially connecting 25 encircled numbers distributed on a sheet of paper.  -Task requirements are similar for TMT-B except the person must alternate between numbers and letters. | Riggio et al., 2011. |
| TMT A/B | Healthy cognitive states:  -TMT A measures the time participants need to connect 25 numbered circles in an ascending order.  -TMT B 13 numbers and 12 letters have to be alternately connected in their numerical and alphabetical order. | García-Martínez et al., 2011; Malaguarnera et al., 2011; Campagna et al., 2014; Zarantonello, 2019. |
| WCST-64 | To measure executive function, this test valid in characterizing cognitive dysfunction in a variety of neurological disorders including traumatic brain injury. | Lin et al., 2014a; Lin et al., 2014b. |
| WMT-RT | Includes complex tasks as comprehension, learning, and reasoning, and comprises the following three components: the phonological loop, visuospatial sketchpad, and central executive system. | Zarantonello, 2019. |
|  |  |  |
| Abbreviations: AVLT, Auditory Verbal Learning Test; BDI, Beck Depression Inventory; BDT, Block Design Test; BNT, Boston Naming Test; BTACT, Brief Test of Adult Cognition by Telephone; CASI, Cognitive Abilities Screening Instrument; CRT, Continuous Reaction Time; COWAT, Controlled Oral Word Association Test; DST, Digit Span Test; EncephalApp, EncephalApp Stroop test; HRQOL, Health-Related Quality of Life; ICT, Inhibitory Control Test; JLO, Judgment of Line Orientation; K-BNT, Korean-Boston Naming Test; K-MMSE, Korea-Mini Mental State Examination; LF, Letter Fluency Test; LTT, Line Tracing test; MMSE, Mini-Mental State Examination; MRT, Mental Rotation Test; NCT-A, Number Connection Test A; NCT-B, Number Connection Test B; PHES, Psychometric Hepatic Encephalopathy Score; PROMIS, Patient-Reported Outcomes Measurement Information System; PRT, Paragraph Recall Test; RBANS, Repeatable Battery for the Assessment of Neuropsychological Status; RCFT, Rey Complex Figure Test and Recognition Trial; SDT, Simple Drawing Test; SDMT, Symbol Digit Modalities Test/Digit Symbol Test/Digit Symbol Coding Test; SF, Semantic Fluency Test; SIPCHE, Sickness Impact Profile Questionnaire for covert Hepatic Encephalopathy; SNSB, Neuropsychological test Seoul Neuropsychological Screening Battery; ST, Stroop Test (Dodrill’s method); SVLT, Seoul-Verbal Learning Test; TMT A, B, Trail-Making Test A and B (Reitan’s method); TMT A/B, Trail Making Test A and B; VOT, Hooper Visual Organization Test; WAIS-III, Wechsler Adult Intelligence Scale/Test; WCST-64, Wisconsin Card Sorting Test Computer Version Scoring Program; WMT-RT, Working Memory Test (ScanRT). | | |
